# Life Cycle Assessment and Life Cycle Costing of Supercapacitors: A Comprehensive Review and Assessment of Environmental and Economic Impacts

**DOI:** 10.1002/cssc.202500583

**Published:** 2025-09-23

**Authors:** Fatemeh Bahmei, Amaia Saenz de Buruaga, Sebastián Pinto Bautista, Javier Olarte, Jon Ajuria, Alberto Varzi, Marcel Weil

**Affiliations:** ^1^ Helmholtz Institute Ulm (HIU) Helmholtzstrasse 11 89081 Ulm Germany; ^2^ BCARE Alava Technology Park 01510 Miñano Alava Spain; ^3^ Institute for Technology Assessment and Systems Analysis (ITAS) Karlsruhe Institute of Technology 76021 Karlsruhe Germany; ^4^ Centre for Cooperative Research on Alternative Energies (CIC energiGUNE) 01510 Vitoria‐Gasteiz Spain; ^5^ Karlsruhe Institute of Technology (KIT) 76021 Karlsruhe Germany

**Keywords:** life cycle assessment, life cycle costing, lithium‐ion capacitors, sodium‐ion capacitors, supercapacitors

## Abstract

Ongoing research on energy storage systems, driven by the energy transition, has led to the development of alternative systems beyond conventional batteries, such as supercapacitors (SCs). These devices offer high‐power density, rapid charge/discharge, and long cycle life, making them suitable for applications requiring quick energy bursts. Moreover, they bear lesser dependency on critical raw materials, enhancing their sustainability. Despite their technological maturity, little is known about their environmental and economic implications from a life cycle perspective. This review offers an insight into life cycle assessment and life cycle costing studies evaluating the environmental impacts and economic viability of SCs. The analysis synthesizes existing research, identifies trends, and highlights key knowledge gaps. By providing a systematic overview of the life cycle sustainability metrics for SC technologies, this study contributes to a deeper understanding of their role as a viable and sustainable energy storage solution. Due to heterogeneous system boundaries and product systems found in literature, a clear estimation of average environmental impacts and cost performance remains challenging. Additionally, the sustainability implications of next‐generation SCs are not fully understood. Further research is needed to establish comprehensive sustainability assessments, improve methodological consistency, and guide future development.

## Graphical abstract

This review synthesizes life cycle assessment and life cycle costing evidence on supercapacitors, highlighting common approaches and gaps. It explains how inconsistent boundaries and functional units limit comparability and propose practical reporting and scenario guidance is proposed to strengthen representativeness and support robust sustainability decisions.

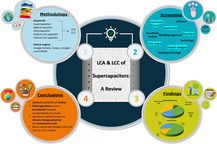

## 1 Introduction

In a world increasingly focused on sustainability, energy storage technologies play a pivotal role in the transition to cleaner, more resilient, and efficient energy systems. In a lithium‐ion battery (LIB) dominated market, electrochemical capacitors (ECs), commonly known as supercapacitors (SCs), have emerged as a promising complementary solution due to their high‐power density, rapid charge/discharge capabilities, long cycle life, and reliable performance across a wide temperature range.^[^ ^1^ ^]^ This ability to deliver bursts of energy in milliseconds makes them ideal for power backup systems, regenerative braking in electric vehicles, and industrial applications where sudden power surges are needed.^[^ ^2^ ^]^

**Figure 1:**
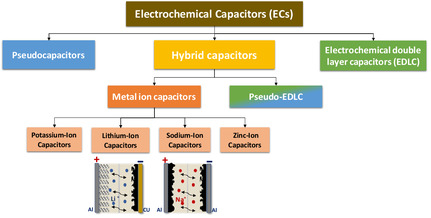
Overview of ECs: classification and types.

ECs are broadly classified into three main categories based on their charge storage mechanisms: pseudocapacitors, electrochemical double‐layer capacitors (EDLCs), and hybrid capacitors (Cap)^[^ ^3^ ^]^ (**Figure** **1**). Pseudocapacitors store energy via fast, reversible redox reactions at the electrode surface, often utilizing materials like transition metal oxides.^[^ ^4^ ^]^ In contrast, EDLCs store energy through the electrostatic separation of charges at the interface between an electrolyte and a porous electrode, with carbon‐based materials predominating due to their high surface area, which facilitates efficient and rapid charge accumulation.^[^ ^5^ ^]^

Unlike LIBs, EDLCs can operate efficiently in extreme temperatures, making them suitable for aerospace, military, and remote sensor applications.^[^ ^6^ ^]^ Additionally, their exceptional cycle stability, often exceeding a million cycles, allows them to operate in scenarios demanding frequent charge–discharge cycles, such as grid stabilization, renewable energy storage, and Internet of Things devices requiring reliable energy buffering.^[^ ^7^ ^]^ These characteristics are enabled by their operation mechanism, which is based on the physical absorption of charges, rather than diffusion limited chemical reactions. Their commercial development began in the 1970s and 1980s, with major advancements in the 1990s as companies like Maxwell Technologies (now part of Tesla) and Nippon Chemi‐Con started producing them at scale. The 2000s and 2010s saw increased adoption, particularly in applications requiring high‐power density and fast charge‐discharge cycles, such as regenerative braking in hybrid buses, power backup systems, and industrial energy buffering.^[^ ^5^, ^8^ ^]^ Nowadays, companies like Skeleton Technologies, Eaton, and Tesla (following its acquisition of Maxwell) are pushing advancements in materials and manufacturing.

Hybrid Cap combine the mechanisms of both pseudocapacitors and EDLCs, achieving a balance between electrostatic and faradaic charge storage.^[^ ^4^, ^9^ ^]^ This hybrid functionality allows pseudo‐EDLCs to achieve higher energy densities than conventional EDLCs.^[^ ^10^ ^]^ In particular, metal‐ion capacitors (MICs) represent a promising category of hybrid Cap due to their ability to combine electrostatic charge storage with the intercalation of metal ions.^[^ ^11^ ^]^ MICs, including lithium‐ion capacitors (LICs), sodium‐ion capacitors (SICs), and potassium‐ion capacitors, offer significantly higher energy densities compared to traditional EDLCs.^[^ ^12^ ^]^

Among these, SICs have garnered significant attention as a promising and cost‐effective alternative to LICs.^[^ ^7^ ^]^ SICs leverage the energy storage potential of sodium ions, which are more abundant and less expensive than lithium.^[^ ^13^ ^]^ This makes SICs particularly attractive for large‐scale applications where cost‐effectiveness and sustainability are key considerations. The shift toward more abundant materials like sodium and potassium seeks not only to improve performance but also to align with sustainability goals by reducing the environmental impact of energy storage systems (ESS).^[^ ^14^ ^]^

A key aspect of the sustainability of SCs lies in balancing costs and environmental impacts across all stages of their life cycle. Material selection, for instance, influences production costs and emissions with electrode materials such as activated carbon (AC) offering advantages in terms of availability and recyclability compared to critical metals used in conventional batteries.^[^ ^15^ ^]^ During operation, their long lifespan and high energy efficiency reduce the costs and waste associated with frequent replacements, providing an economic advantage over other storage technologies. Finally, at the end‐of‐life (EoL) stage, the potential for material recovery not only reduces environmental impacts but can also generate economic savings through reintegration into new production cycles.^[^ ^16^ ^]^

To comprehensively evaluate these aspects, systematic assessment methods such as life cycle assessment (LCA) and life cycle costing (LCC) are essential. LCA evaluates environmental impacts from raw material extraction to EoL, including categories such as carbon footprint, resource consumption, and waste generation. Meanwhile, LCC assesses cumulative costs across production, operation, and EoL stages.^[^ ^17^, ^18^ ^]^ These methods provide insights into how design choices and material selection influence both economic feasibility and environmental performance. Despite the potential advantages of SCs, LCA research remains significantly limited compared to LIBs. Existing studies predominantly focus on precursor carbon material production, whereas LIB research extends to the full system‐level analysis. This scarcity of holistic assessments is even more pronounced for next‐generation SCs like LICs and SICs, highlighting a crucial research gap.

In this context, this article presents a comprehensive review of the current literature on the LCA and LCC of SCs, encompassing all generations from traditional EDLCs to emerging technologies like SICs. Our analysis synthesizes existing findings, highlights methodological trends, and assesses the overall landscape of research on life cycle sustainability metrics for SC technologies.

Furthermore, this article aims to identify key areas requiring further research, especially for next‐generation SCs like LICs and SICs, where LCA studies are notably scarce. By highlighting these gaps, we seek to stimulate future research directions that will contribute to a more comprehensive understanding of the sustainability profile of SCs.

## 2 Review Methodology

An extensive literature review has been conducted to identify available studies related to the sustainability character of SCs and hybrid Caps, in particular studies that provide a quantitative analysis of SCs’ environmental profile and cost efficiency, that is, LCAs and LCCs, respectively. The search for studies was carried out, in particular for LCAs, via the search engines Google Scholar, Scopus, and the Community Research and Development Information Service (CORDIS), and for LCCs, via Google Scholar, Google, and CORDIS. The difference between the two groups lies in the fact that, presumably, some economic assessments are more likely to be published on commerce‐oriented platforms, found on Google, instead of entirely academic portals. Google Scholar and Scopus were considered sufficient for the identification of academic literature on an international level, whereas the CORDIS platform may provide further information arising from projects funded by the European Commission, which may be presented in the form of reports instead of journal publications.

**Figure 2:**
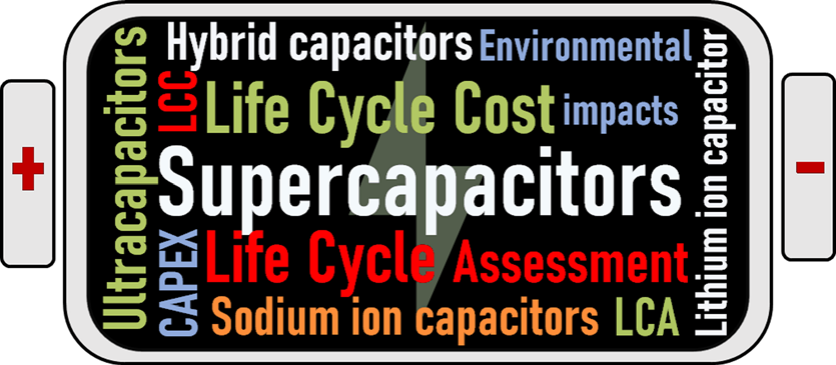
Visualization of keywords.

Similarly, two different sets of keyword strings were defined. For the identification of LCAs, the strings “SC AND (LCA OR environmental impacts)”, “hybrid Cap AND (LCA OR environmental impacts)”, “ultracapacitor (UC) AND (LCA OR environmental impacts)”, ‘SIC AND (LCA OR environmental impacts)”, and “LIC AND (LCA OR environmental impacts)” were used. For LCCs, the keyword strings “SC AND (LCC OR cost OR CAPEX)”, “hybrid capacitor AND (LCC OR cost OR CAPEX)”, “UC AND (LCC OR cost OR CAPEX)”, “SIC AND (LCC OR cost OR CAPEX)” and “LIC AND (LCC OR cost OR CAPEX)” were used (**Figure** **2**). Studies that provide a quantitative analysis of environmental impacts or cost assessment of Cap production from the year 2010 to 2024 have been considered. Studies about e‐mobility or stationary power systems which include a description of the production of SCs have also been recorded.

The technical and technological aspects registered in each column of **Table** **1** and **2** are considered of most relevance for LCAs and LCCs, respectively, as they provide a sufficient basis for system characterization with a view on comparability. Details of the type of technology, stage of development, intended application, boundaries of the model, source of data, functional unit (FU), and impact assessment method were the base of the screening.

**Table 1:** Main characteristics of LCA studies (scientific articles) on Sc, Cap, and LFP identified by literature search (2010–2023).

Year	Technology	FU	LCA modeling approach	Software and database	Development stage	Sensitivity analysis	Ref.
2012	SC	kg carbon‐based electrode material	Cradle to gate		Lab scale	–	–
2015	SC	one vehicle lifetime of 12 y with a driven distance of 150,000 km	Cradle to grave	Umberto 5.5, ecoinvent 2.2	Industrial	Electricity supply chain	[18]
2018	Cap	1 kg for each electrode with capacitance of 1 μF	Cradle to grave	–	Prospective industrial	Primary energy consumption	[15]
2019	SC	1000 mAh at the current density of 1 A g^−1^ over the lifetime of the electrodes defined by the capacity fade of 20%	Cradle to gate	openLCA 1.5, ecoinvent 3.3	Lab scale	Process efficiency and electrochemical performance	[38]
2019	SC	1 ton of V_2_O_5_ crystals	Cradle to gate	GaBi 8.7, ecoinvent 3.4	Lab scale	–	[27]
2019	SC	1 g Co_3_O_4_ (electrode material)	Gate to gate	SimaPro 8.0.3.14, ecoinvent v 3.1	Lab scale	Electricity supply chain	[36]
2020	SC	One SC rack of five SC with capacitance of 5 F	Cradle to grave	GaBi 7.0	Industrial	Electricity supply chain	[19]
2021	SC	5 F	Cradle to gate	SimaPro	Lab scale	–	[23]
2021	LIC	Cells which make a 48 V LIC module	Cradle to gate	SimaPro, ecoinvent 3.5	Lab scale	–	[24]
2021	SC	one micro SC	Cradle to gate	SimaPro	Lab scale	–	[29]
2022	SC	1 F	Cradle to gate	Open LCA, ecoinvent 3.6	Industrial	–	[22]
2022	SC	One batch of graphene production (electrode material)	Cradle to grave	Simapro 9.1.1.1, ecoinvent 3.	Lab scale	–	[32]
2022	SC	1000 Kg of AC (electrode material)	Cradle to gate	SimaPro 9	Industrial	Recycling of KOH and use of steam activation	[40]
2022	SC	1 g of floc sludge for carbon electrode material	Cradle to gate	eBalance, ecoinvent v3.1	Lab scale	–	[25]
2022	SC	kWh/1 m^2^ & one faced panel	Cradle to gate	openLCA 1.10.3	Prospective industrial	Different FUs	[20]
2022	Cap	1,000,000 pieces of the AECs with 25 V rated working voltage and 150 μF rated capacitance	Cradle to grave	GaBi 10.6.1.35	Industrial	Electricity supply chain and primary aluminum production	[33]
2022	Cap	100,000 high‐voltage AECs (420 V, 680 μF), each with 3000 operating hours	Cradle to grave	GaBi 10.6.1.35	Prospective industrial	A 5% increase from baseline for electricity used in production, aluminum ingot mass for anode foil, aluminum ingot mass for cathode foil, design capacitance, and electrolyte mass.	[34]
2023	SC	1 F supplied at 3.5 V, 1 W supplied for 1 min and 1 Wh supplied in 1 min	Cradle to gate	openLCA 1.11.0, ecoinvent 3.8	Prospective industrial	–	[28]
2023	SC	1 kg of PANI/GO nanocomposite (electrode material)	Cradle to gate	openLCA	Lab scale	–	[26]
2023	SC	1 kg of NTO (electrode material)	Gate to gate	SimaPro 9.0.0.29, ecoinvent 3.1	Lab scale	–	[35]
2024	SC	1 kg of AC and 1 farad (F) of electrode	Cradle to gate	Simapro 9.4.0, Recipe midpoint, ecoinvent 3.6	Lab scale	Focus on wastewater generation and electricity consumption during hydrothermal carbonization	[37]
2024	SC	kg of LAC, activated biochar (electrode material)	Cradle to gate	GaBi ts 10.7, ecoinvent 3.8 and AGRIBALYSE	Lab scale	Energy recovery, KHCO_3._ and ethanol recovery scenarios)	[43]
2024	Dual ESS with Li‐ion, aqueous Al‐ion, and SC	Per dual ESS	Cradle to gate	OpenLCA 1.10.3, Ecoinvent 3.2	Lab scale	Transport distances, electricity mix, and material recycling scenarios	[46]
2024	SC	Ton of AC (electrode material)	Cradle to gate	ReCiPe 2016 midpoint (H)	Prospective industrial	AC price, raw material costs, utility costs, tax rates, plant capacity, and operating hours	[44]

**Table 2:** Main characteristics of LCC studies on SCs identified by literature search (2010–2024).

Year	Technology	FU	Development stage	Application	Boundary	Cost analysis	Ref.
2010	Batteries, UC, and FC	$	State‐of‐the‐art review	Mobility	Battery pack	Cost	[49]
2012	SC	£	Unit market prize	Mobility	Battery pack	–	[68]
2013	SC	$ kg^−1^	Component market prize	Not specified	Electrode material	–	[58]
2013	SC	€ kW^−1^	Early stage	Mobility	Cell	Cell production cost	[17]
2014	UC	$	Simulation	Mobility	Entire system	Cash flow in 12 years	[72]
2015	LIB, SC	$ kW^−1^, $ kWh^−1^	Model	Renewable energy integration	Cell	Capital, O&M, and replacement costs	[67]
2015	Battery, UC, LIB, FC	€, kg per €	Simulation	Mobility	Entire system	CAPEX, running costs, and payback times	[73]
2016	SC, batteries	Parameter/%	Simulation	Renewable energy integration	Battery pack	Current gain, energy losses, and global efficiency per cost (%)	[2]
2016	Pb‐acid, SC, LFP	$ month^−1^, savings (%)	Simulation	Stationary	Battery pack	TCO	[74]
2016	Battery, SC	$ kWh^−1^	Simulation	Stationary	Entire system	Annual revenue, cost, and profit	[75]
2016	SC	PLN year^−1^	Existing and planned	Mobility	Entire system	Cost of investment, maintenance, and scrapping costs	[70]
2016	SC, LTO	€ day^−1^	Existing and proposed solution	Mobility	Battery pack	Operating costs	[76]
2018	SC, battery	$	Simulation	Renewable energy integration	Entire system	Initial cost, replacement cost, and O&M cost.	[53]
2018	FC, SC	$ kW^−1^	Simulation	Renewable energy integration	Battery pack	Capital, replacement, and O&M, salvage	[47]
2018	Pb‐acid, SC	$ kWh^−1^, %	Simulation	Renewable energy integration	Battery pack	LCOE, CAPEX, reinvestment, and O&M	[60]
2018	Pb‐acid, LIB	$		Stationary	Battery pack	Average total cost, amortization CAPEX, OPEX	[77]
2018	SC and batteries	$ km^−1^, $	Simulation	Mobility	Entire system	Battery price, capital cost, electricity cost, and replacement cost	[57]
2019	SC, battery	$, $ kW‐year^−1^	Simulation	Renewable energy integration	Entire system	CAPEX, fixed O&M, fuel cost, and electricity cost	[69]
2019	Pb‐acid, FC, LIB, SC	$ kWh^−1^	Simulation	Renewable energy integration	Entire system	Total equipment and installation costs, total O&M costs, total replacement costs	[61]
2019	LFP, nickel manganese cobalt, SC	$	Simulation	Mobility	Battery pack	Initial and operational costs	[50]
2019	LIB, redox flow battery (RFB), Pb‐acid, sodium–sulfur (NaS), Na metal halide, zinc hybrid, pumped storage, flywheels, compressed‐air energy storage, UC	kW, kWh	Market	Not specified	Entire system	CAPEX, O&M	[62]
2020	LIB, RFB, Pb‐acid, NaS, UC…	$ kW‐year^−1^, $ kW^−1^, $ kWh^−1^	–	Stationary	Entire system	CAPEX (with cost breakdown), fixed OPEX	[65]
2020	Battery, SC	€	Simulation	Mobility	Entire system	SC and powertrain modification cost (€), total battery cost, and forklift EoL (€), total cost of storage system (€) and LCC reduction (%)	[78]
2020	LIB, SC, LIC	€ kW^−1^, € kWh^−1^	Market	Mobility	Cell	CAPEX	[64]
2020	UC, LIB	$ kWh^−1^	Industry	Not specified	Cell	Cost	[66]
2020	SC	kW	Technology readiness levels (TRL)9	Stationary	Battery pack	Initial cost, maintenance and replacement cost, disposal cost	[55]
2021	LIB, SC	kWh	Simulation	Renewable energy integration	Battery pack	ESS cost	[52]
2021	LIB, Pb‐acid, SC	$ kWh^−1^	Simulation	Renewable energy integration	Entire system	Base cost, converter base cost, inverter base cost	[79]
2021	SC, LIB, flywheel	kW	TRL9	Stationary	Battery pack	Efficiency cost, maintenance cost, and CAPEX	[56]
2021	Batteries, SC, flywheels	200 kW		Stationary	Battery pack	Initial cost, annual cost, maintenance, and replacement	[80]
2022	SC	USD	Industrial	Not specified	Electrode material	CAPEX, OPEX, and NPV	[40]
2023	SC	kW, kWh	Market	Not specified	Entire system	CAPEX, O&M	[81]
2023	SC	Yuan	Simulation	Renewable energy integration	Cell	Purchase, operating, maintenance, and disposal (salvage cost and EoL) cost of the equipment	[63]
2023	LIB, SC	€, € MW^−1^, € year^−1^	Market	Stationary	Cell	CAPEX, OPEX	[54]
2024	SC	USD kg^−1^, USD kWh^−1^, USD m^−3^, %, USD year^−1^	Lab scale	Not specified	Electrode material	NPV, CAPEX	[44]

## 3 Literature Review Results

### 3.1 LCA

**Figure 3:**
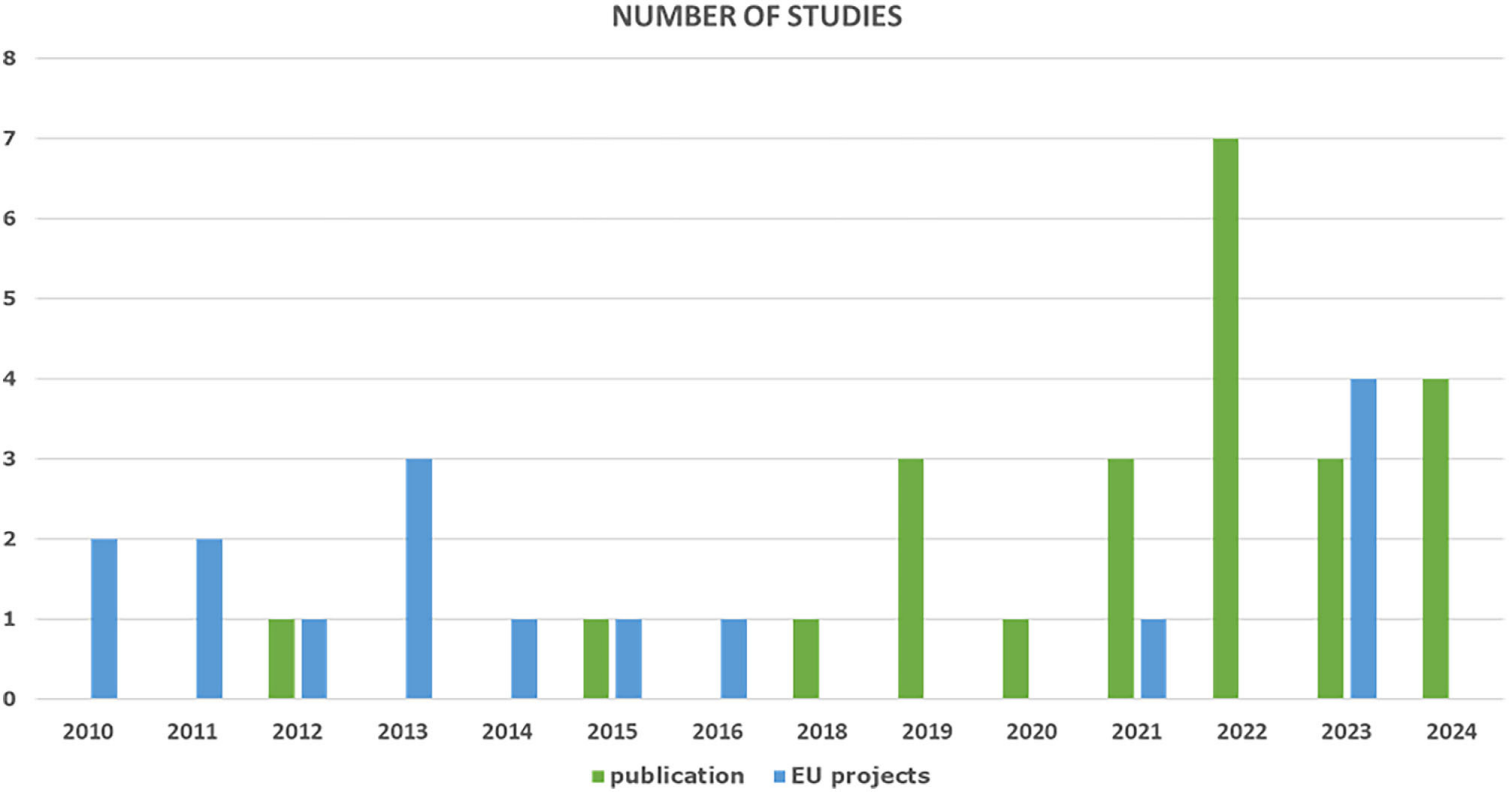
Number of LCA publications and EU‐projects per year between 2010 and 2024.

The review included a diverse selection of academic articles, reports, and other relevant publications, totaling 40 sources (**Figure** **3**). Notably, this comprehensive examination transcends traditional research publications, and incorporates insights derived from projects funded by the European Union (EU's) framework programs for research and innovation. In particular, a total of 16 EU‐projects were identified, which focused either on the development of new SC technologies or on the development of novel materials for SC applications, with attention to the sustainability profile of the technologies assessed (according to the reported project description). However, 10 of the 16 projects did not provide publicly available literature regarding LCA or environmental assessments. Of the three projects which did provide this information, one (AUTOSUPERCAP) resulted in a publication already identified via the academic portals. Another (NETFICCIENT) provided a superficial analysis of environmental impacts from SCs used for renewables support. The third (HYHEELS) provided a comparative assessment of SC versus different battery systems in automotive applications. Four other projects (EMPHASIS, GREENCAP, MUSIC, and HEDAsupercap), which started in 2023, have not yet published any results regarding LCA or environmental assessments.

#### 3.1.1 Goal and Scope

The goal and scope defined in LCA studies determine the purpose, the system boundaries and the FUs. This serves to define, for instance, which product system and life cycle stages are to be considered as well as the system level to be analyzed and operation parameters. Among the 24 reviewed articles, it was noted that 13 predominantly focused on studying the production of electrode materials as a product system. Only four articles specifically addressed the application/use‐phase of SCs. For instance, in 2015, Weil et al. conducted a LCA of SCs in electric vehicles.^[^ ^18^ ^]^ Cossutta et al. in 2020 delved into a comparative LCA of graphene and AC in a SC application. Their focus was on evaluating the performance of SCs designed for Fiat Chrysler Automobiles car mirrors.^[^ ^19^ ^]^ In 2022, Hatzfeld et al. conducted research investigating the LCA of SCs. evaluating the environmental impacts of the entire SC device as part of a more extensive system.^[^ ^20^ ^]^

Concerning system boundaries, 16 studies^[^ ^20^, ^21^, ^22^, ^23^, ^24^, ^25^, ^26^, ^27^, ^28^, ^29^, ^30^, ^31^ ^]^ adopted a cradle‐to‐gate perspective, emphasizing the environmental impact of raw materials extraction until completion of the manufacturing process. Six articles^[^ ^15^, ^18^, ^19^, ^32^, ^33^, ^34^ ^]^ adopted cradle‐to‐grave boundaries, encompassing the entire life cycle from production to disposal. Additionally, two studies^[^ ^35^, ^36^ ^]^ implemented a gate‐to‐gate perspective, concentrating specifically on the impacts of the manufacturing processes without considering the broader life cycle stages.

While a cradle‐to‐gate system boundary is common and valuable for understanding the environmental impact of supply chain and manufacturing phases, it is essential to acknowledge that a complete LCA, covering the entire life cycle, provides a more holistic view of the sustainability of products. The choice of system boundaries, although dependent on the study's goal and data availability, can significantly affect the representativeness of the results. For instance, studies that adopt a cradle‐to‐gate perspective may overlook critical impacts that occur during the use‐phase and the EoL disposal or recycling of SCs, leading to an incomplete understanding of the total environmental footprint of the product. The use‐phase may involve energy consumption and emissions that are not accounted in the earlier life cycle stages of an SC. Poor performance during operation due to factors such as inefficient design or system degradation could overshadow the intrinsic environmental advantages of SCs over other systems. Additionally, EoL management involving disposal practices and recycling processes could also play a crucial role in determining the overall sustainability of SCs. Neglecting the environmental implications associated with recycling, influenced by material recovery rates and energy required for reprocessing, may lead to present a skewed view of the product's environmental impact. In essence, by not encompassing the full life cycle from cradle‐to‐grave, researchers risk presenting overly optimistic assessments of sustainability that fail to capture significant environmental hotspots.

To improve the accuracy and relevance of LCAs for SCs, it is essential for future studies to adopt a comprehensive life cycle approach that includes all phases, from extraction and manufacturing through use and final disposal or recycling. This holistic perspective will facilitate a more accurate understanding of the sustainability performance of SCs and support better decision‐making in their design and application.^[^ ^37^ ^]^ With respect to the FU, the selection of a FU in the LCA of ESS can vary, contingent on the study's goals, scope, and the specific characteristics of the energy storage technology under evaluation. Common FUs in the LCA of ESS include units of energy delivered, storage capacity, capacitance, power capacity, lifetime energy throughput, and other units tailored to the specific application of the ESS. Among the reviewed studies, Cossutta et al.,^[^ ^19^ ^]^ Jiang et al.,^[^ ^38^ ^]^ Glogic et al.,^[^ ^22^ ^]^ and Kamali et al.^[^ ^39^ ^]^ defined the FU in terms of capacitance. In the study by Cossutta et al., for instance, the FU was defined as one SC rack consisting of five SC units, each with a capacitance of 5 F.^[^ ^19^ ^]^ When focusing on the electrode, Glogic et al. established a FU of 1 F,^[^ ^22^ ^]^ whereas Jiang et al. specified an FU of 5 F.^[^ ^23^ ^]^Other nine studies defined the FU in terms of mass of electrode material.^[^ ^21^, ^25^, ^26^, ^27^, ^35^, ^40^ ^]^ Wang et al.^[^ ^40^ ^]^ defined the FU as production of 1000 kg of AC for the electrode. De et al. set the FU as production of 1 kg of Na_2_Ti_3_O_7_ as electrode material for SICs.^[^ ^35^ ^]^

Mass‐based FUs are useful for comparing production methods and screening electrode materials, especially at early development stages. In contrast, service‐based FUs (e.g., Wh delivered under defined voltage, temperature, duty/power profile, and lifetime; or capacitance linked to electrolyte, resistance limit, test protocol, and voltage range) are needed to represent device or application performance. For results to be comparable across studies, authors should report the FU operating conditions (voltage range, electrolyte, temperature, duty cycle, and EoL criterion). Without this information, values expressed only per F or per Wh cannot be reliably compared.^[^ ^41^ ^]^

This approach provided a comprehensive evaluation from raw materials extraction to the final fabrication of these critical components, recognizing the pivotal role that electrode materials play in SC performance. However, the variability in FUs across studies hampers comparability and can lead to differing conclusions regarding sustainability metrics, emphasizing the need for standardized definitions in future research. Table 1 provides an overview of the other FUs that are used in the studies. Future researchers should consider adopting a standardized framework for defining system boundaries and FUs, as this will enhance the comparability of LCA results across different studies.

#### 3.1.2 Data Sources

In LCA, data sources play a pivotal role to ensure the reliability and robustness of the results. These sources can be broadly categorized into primary and secondary data. Primary data are collected specifically for the study, for example through surveys, experiments, or direct observations. Secondary data encompasses preexisting information from sources such as literature, databases, or previously conducted LCAs. Secondary datasets are often employed to increase efficiency and reduce costs, particularly for modeling background processes such as electricity mixes and supply chains.

The advantage of primary data lies in its specificity to the research question, ensuring relevance and accuracy. Secondary data, while more readily available and cost‐effective, may lack the precision of primary data. Moreover, secondary sources are most commonly used to model processes in the background, such as electricity mix and supply chains. In the present review, 23 studies were identified as incorporating primary data into their LCA. Most of these focused on assessing the environmental impacts associated with electrode‐material production. Examples include the works of Weil et al.,^[^ ^21^ ^]^ Sharma et al.,^[^ ^36^ ^]^ Li et al.,^[^ ^27^ ^]^ Glogic et al.,^[^ ^38^ ^]^ Wang et al.,^[^ ^40^ ^]^ Zhang et al.,^[^ ^33^ ^]^ and De et al.^[^ ^35^ ^]^ In these studies, primary data was normalized according to a FU of mass of material produced, ranging from grams of Co_3_O_4_ in Sharma et al.'s study^[^ ^36^ ^]^ to the production of 1000 kg of AC as an electrode material in the research by Wang et al.^[^ ^40^ ^]^

In the studies by Jiang et al.^[^ ^23^ ^]^ and Glogic et al.,^[^ ^22^ ^]^ primary data for electrode production were collected and normalized to a FU of 1 and 5 Farads, respectively. Glogic et al.^[^ ^22^ ^]^ collected primary data on energy consumption and material inputs during lab‐scale production of AC electrodes derived from coconut shells, and additionally modeled a hypothetical industrial‐scale scenario to compare environmental impacts across scales. Similarly, Cossutta et al.^[^ ^19^ ^]^ compared the environmental performance of graphene and AC electrodes using primary data from both lab‐scale and simulated industrial production. This dual approach provided insights into scaling effects on environmental impacts.

In contrast, studies such as Zhang et al.^[^ ^33^ ^]^ focused exclusively on laboratory‐scale data for floc sludge‐derived electrodes, limiting the assessment to the production in early design‐phase without addressing use‐phase or EoL impacts. Hatzfeld et al.^[^ ^20^ ^]^ adopted a broader perspective, conducting a cradle‐to‐site LCA of a multifunctional facade integrating SCs, based on both laboratory and pilot‐scale prototypes. This study underscored the importance of scaling from prototypes to real‐world applications, providing a more realistic assessment of potential environmental impacts and benefits of the novel technology when implemented at a commercial scale, rather than limiting the analysis to small‐scale prototypes. In the research conducted by Zimmerman et al.,^[^ ^18^ ^]^ Cossutta et al.,^[^ ^19^ ^]^ Chigada et al.,^[^ ^24^ ^]^ Li et al.,^[^ ^29^ ^]^ Dericiler et al.,^[^ ^32^ ^]^ and Hatzfeld et al.^[^ ^20^ ^]^ primary data for a SC components, including electrodes (positive and negative), separator, electrolyte, current collector, and others, were considered in the LCA.

Complete life cycle inventories (LCIs) were accessible for 18 of the studies that relied on primary data. In terms of software, approximately two‐thirds of the reviewed studies used SimaPro, openLCA, and GaBi as their LCA software. One study used Umberto,^[^ ^18^ ^]^ and another utilized eBalance software.^[^ ^25^ ^]^ In one article, no details were provided about the specific software used.^[^ ^15^ ^]^

#### 3.1.3 Impact Assessment Methodology

**Figure 4:**
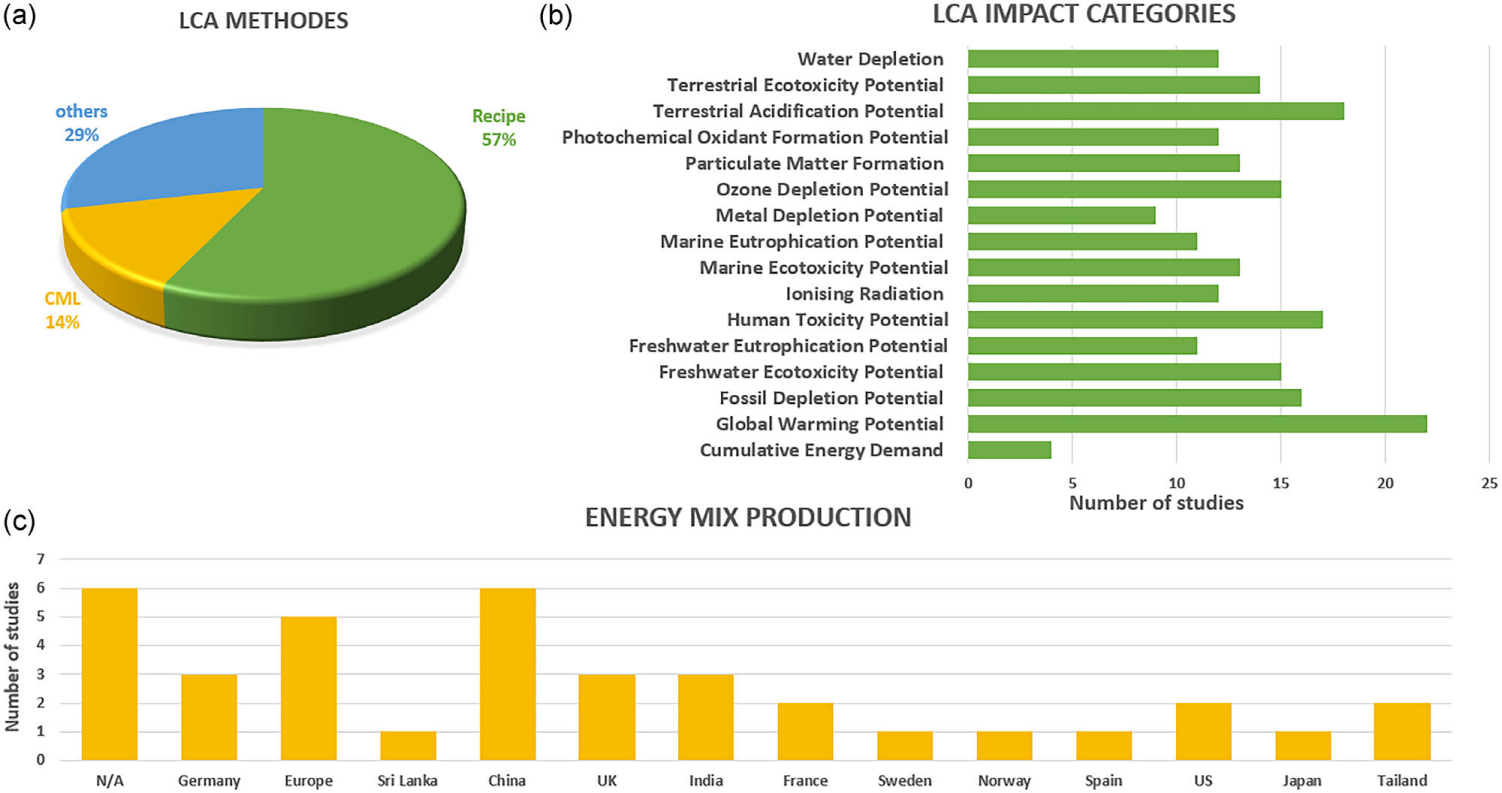
The LCA literature review: a) the LCA methods assessed, b) the impact categories in the selected LCA studies, and c) the number of studies per electricity mix (region).

A wide variety of impact assessment methods were applied across the reviewed studies, with the choice of method largely depending on the specific goals of the study. Among the reviewed studies, ReCiPe was the most frequently applied, used in ≈57% of cases (12 studies), followed by the CML method, employed in three studies. Other methods included ILCD, USEtox, IMPACT 2002+, and TRACI (**Figure** **4**a). Each method addresses a distinct set of environmental concerns, providing complementary perspectives on the potential environmental impacts of products or processes. For example, the ReCiPe methodology covers 18 impact categories, including climate change, ozone depletion, human toxicity, ecosystem quality, and resource depletion, among others.^[^ ^42^ ^]^ Among the reviewed studies, global warming potential (GWP) emerged as the most commonly reported impact category, assessed in 22 studies. Other frequently evaluated categories included terrestrial acidification potential (18 studies), human toxicity (17 studies), and fossil depletion potential (16 studies). Additional impact categories identified in the review are summarized in Figure 4b. The diversity of methods used in literature poses challenges for cross‐study comparability. Developing a standardized framework for the assessment of SCs and their components would help address this issue, enabling more consistent benchmarking and system comparisons under harmonized criteria for evaluating of their environmental performance.

#### 3.1.4 Environmental Impact of SCs

##### 3.1.4.1 Challenges in Comparing Findings from the Literature

To draw insights from the state‐of‐the‐art in LCA of SCs, it is essential to compare the approaches and findings reported in the literature. Such comparisons allow the estimation of average values and the derivation of meaningful conclusions regarding environmental impacts, efficiency, and potential applications. However, several challenges limit comparability across studies. One major issue is the wide range of functional or reference units (FUs) employed, including capacitance (F), stored energy (kWh), mass of material produced (kg), and specific capacity (mAh). In principle, FUs could be converted to a unified unit, but the data reported in most studies are insufficient to enable such harmonization. These discrepancies in FUs, alongside differences in product systems and system boundaries, reflect the diversity of technologies and applications considered.

For example, some studies focus solely on the synthesis of active electrode materials, calculating impacts per kilogram of material produced (e.g., kg of graphene,^[^ ^32^ ^]^ Na_2_Ti_3_O_7_,^[^ ^35^ ^]^ or AC^[^ ^40^ ^]^). Other studies analyze specific applications of SCs, such as their use for electromobility in Germany (FU = 1 vehicle lifetime of 12 years with a driven distance of 150,000 km)^[^ ^18^ ^]^ or multifunctional facades (FU = kWh m^−2^).^[^ ^20^ ^]^ Beyond FU inconsistencies, differences in life cycle impact assessment methodologies further complicate comparability. Studies report results across varying impact categories (Figure 4a,b), though most studies at least discuss GWP, with some extending to additional impact categories.

Beyond differences in scope (e.g., cradle‐to‐gate vs. cradle‐to‐grave), variations in manufacturing conditions could introduce further discrepancies. Results can be largely influenced by the energy mix used in production processes and by the production scale at the manufacturing plant. Figure 4c displays the electricity grids used in SC production across studies. The most frequently used source of energy is the Chinese grid (six studies), followed by Germany, Europe (average), India, and Sri Lanka. However, six studies did not specify an electricity mix or focused only on cumulative energy demand (CED). With respect to production scales, mass, and energy flows in laboratory‐scale environments could differ several orders of magnitude from those in industrial‐ or pilot‐scales.^[^ ^42^ ^]^ Of the reviewed studies, 11 focused on laboratory‐scale SC production, while 10 examined industrial or prospective industrial production. These differences must be accounted for in comparative assessments. Given these variations in system boundaries, FUs, and methodologies, it remains difficult to derive a meaningful global picture of the environmental impacts of SCs. Greater harmonization of LCIs is required. Establishing consistent system boundaries and FUs would enable fairer comparisons and a more comprehensive understanding of the environmental impact of SCs.

##### 3.1.4.2 LCA of AC for SCs

**Figure 5:**
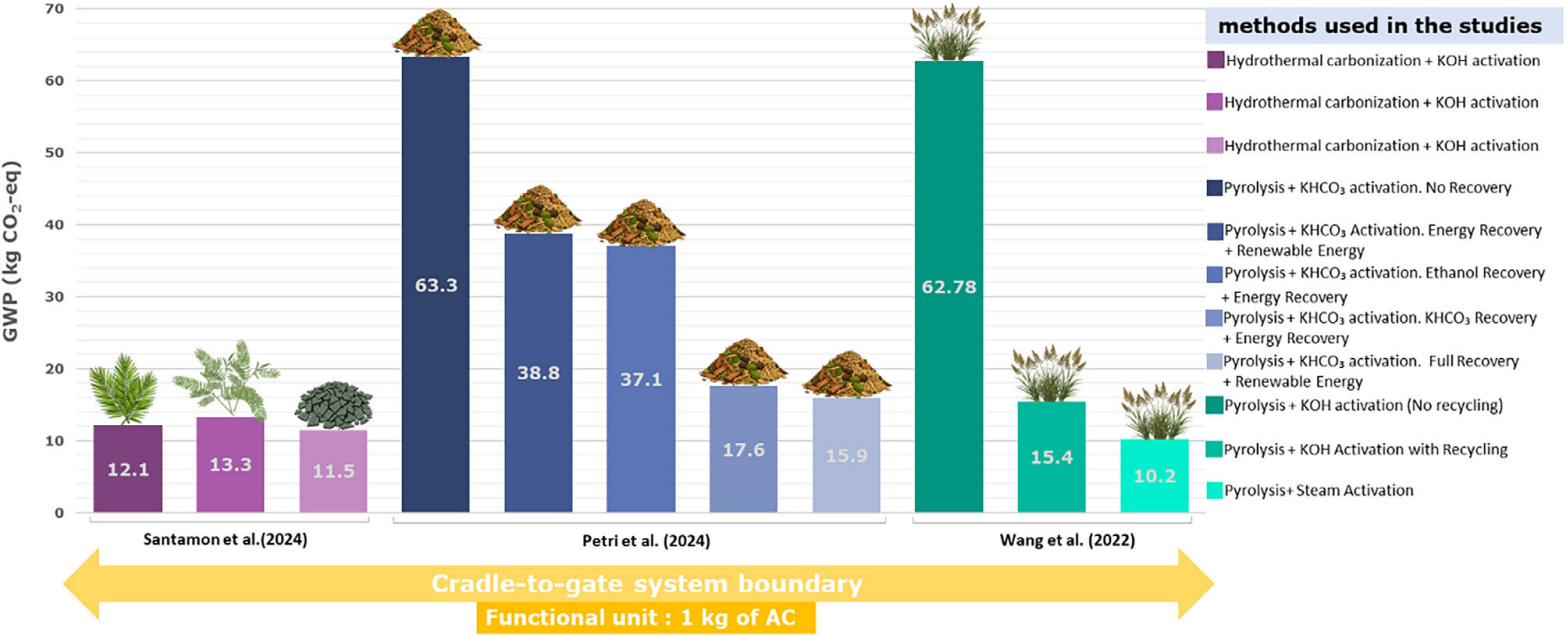
Comparison of GWP for 1 kg of AC across various production methods, based on cradle‐to‐gate analyses as reported in the literature.

Another example of how variations in FUs and system boundaries affect comparability can be found within the assessment of AC as an electrode material. AC has been extensively investigated as an electrode material for SCs. Six studies^[^ ^21^, ^25^, ^37^, ^40^, ^43^, ^44^ ^]^ on this topic have employed a cradle‐to‐gate system boundary to assess the environmental impacts of AC production. Among them, four studies normalized their data per kilogram of AC produced as an electrode material. Three studies reported outcomes specifically for GWP,^[^ ^37^, ^40^, ^43^ ^]^ and one study provided results exclusively for CED.^[^ ^21^ ^]^ **Figure** **5** illustrates the comparison of GWP for 1 kg of AC across various production methods, based on cradle‐to‐gate assessments,

The studies by Luanwuthi et al.,^[^ ^37^ ^]^ Petri et al.,^[^ ^43^ ^]^ and Wang et al.^[^ ^40^ ^]^ employed a cradle‐to‐gate LCA approach to assess the environmental impacts of AC production at different scales. Samanta et al. focused on laboratory‐scale production of AC electrodes from waste materials, using hydrothermal carbonization and chemical activation. Petri et al.^[^ ^43^ ^]^ investigated AC production obtained via pyrolysis, activation, and purification, identifying environmental hotspots, and recommending process optimizations. Their LCI data were upscaled using the framework by Piccinno et al.^[^ ^45^ ^]^ Wang et al. examined industrial‐scale production of highly porous AC from lignocellulosic biomass, analyzing both environmental and economic impacts across the supply chain, from feedstock establishment to in‐plant processing.^[^ ^40^ ^]^

##### 3.1.4.3 Influence of Precursor Selection

The choice of precursor material plays a critical role in both environmental impact and electrochemical performance. For instance, Luanwuthi et al.^[^ ^37^ ^]^ compared oil palm leaves, Sesbania, and filter cake as precursors, finding that oil palm leaves exhibited the lowest GWP of 12.1 kg CO_2_‐eq kg^−1^ AC due to their high specific capacitance (237.6 F g^−1^), which reduces material demand. In contrast, filter cake achieved the lowest GWP per kilogram of AC (11.5 kg CO_2_‐eq) but exhibited poor electrochemical performance (7.3 F g^−1^), leading to higher material and energy demands per FU of 1 F. Similarly, Petri et al.^[^ ^43^ ^]^ used lignin‐rich biodigester waste and demonstrated that process optimizations, such as KHCO_3_ recovery and energy recovery, significantly reduced GWP by up to 75% compared to the base case.

##### 3.1.4.4 Impact of Activation Methods

Activation methods significantly affect environmental impacts, particularly chemical activation using KOH or KHCO_3_. Wang et al.^[^ ^40^ ^]^ reported that KOH activation without recycling resulted in a high GWP of 62,780 kg CO_2_‐eq ton^−1^ AC, whereas recycling KOH reduced this value by 75% (15,400 kg CO_2_‐eq ton^−1^ AC). Steam activation, which eliminates the need for hazardous chemicals, achieved the lowest GWP (10,200 kg CO_2_‐eq ton^−1^ AC) but slightly reduced production efficiency.^[^ ^40^ ^]^ Zhang et al. (2022) highlighted the environmental trade‐offs of acid pretreatment and KOH activation, showing that HNO_3_‐treated sludge combined with a KOH/C ratio of 3:1 produced AC with excellent electrochemical performance (287 F g^−1^) and reduced resource consumption and toxicity risks by over 90% compared to traditional sludge disposal methods.^[^ ^25^ ^]^

##### 3.1.4.5 Process Optimization for Sustainability

Process optimizations, such as energy recovery, chemical recycling, and the use of renewable energy, were identified as key strategies to reduce environmental impacts. Petri et al.^[^ ^43^ ^]^ demonstrated that combining KHCO_3_ recovery, ethanol recovery, and energy recovery with 100% renewable energy sources reduced GWP from 63.3 to 15.9 kg CO_2_‐eq kg^−1^ AC, representing the most sustainable pathway. Similarly, Luanwuthi et al.^[^ ^37^ ^]^ and Wang et al.^[^ ^40^ ^]^ emphasized the importance of recycling and energy recovery in minimizing environmental burdens. These findings underscore the need for integrated approaches that combine material efficiency, chemical recycling, and renewable energy to enhance the sustainability of AC production for SC applications.

#### 3.1.5 Sensitivity Analysis

A sensitivity analysis quantifies the variation in results when model assumptions or conditions change. By doing so, it becomes possible to identify the system elements or parameters with the largest influence on the total impacts. This understanding increases robustness and reliability of the study and provides vital information for stakeholders and decision‐making processes. In the reviewed literature, 45% of the studies conducted sensitivity analyses related to SCs. Several assessed potential changes in the energy mix used for production and use phases, including Zimmerman et al.,^[^ ^18^ ^]^ Cossutta et al.,^[^ ^19^ ^]^ Sharma et al.,^[^ ^36^ ^]^ Melzack et al.^[^ ^46^ ^]^ For instance, in their LCA study of graphene and AC in SC applications, Cossutta et al.^[^ ^19^ ^]^ demonstrated that substantial reductions in greenhouse gas (GHG) emissions associated with SC manufacturing and use can be achieved as a result of changes in the energy mix for electricity generation. This was illustrated by substituting the baseline EU electricity mix with the Norwegian electricity mix in their model. The research conducted by Wang et al.^[^ ^40^ ^]^ which delves into the LCA of AC production from lignocellulosic biomass as electrode material, concludes from a sensitivity analysis that the use of KOH (potassium hydroxide) without recycling not only results in a significant environmental impact in terms of GHG emissions but also poses risks to human toxicity. Notably, wastewater containing KOH in an aqueous mix is identified as a potential source of hazardous waste. Consequently, the recovery of KOH for reuse emerges as a viable strategy, offering both economic and environmental benefits to AC production. Table 1 provides details about sensitivity analyses in the other studies. Overall, these sensitivity analyses highlight the critical influence of key parameters, such as energy sources and chemical inputs, on the environmental performance of SC production.

### 3.2 LCC

In order to make information on SC LCCs more accessible, a comprehensive review of the relevant academic and industry was conducted, encompassing 41 sources and including a varied selection of academic papers, global and market reports, and other relevant publications. This comprehensive review goes beyond traditional research publications, as it incorporates insights derived from projects funded by the EU research and innovation framework programs as well as information from the technology manufacturers themselves. Of the 41 sources examined, only 34 publications provide sufficient data for analysis.

Studies on LCC mainly address cost comparisons between systems that rely predominantly on fossil fuels (or that lack energy recovery integration) and novel systems that incorporate SCs for energy storage. Yet, available information on the economics of SCs, as well as LICs and SICs, remains scarce or altogether absent.

**Figure 6:**
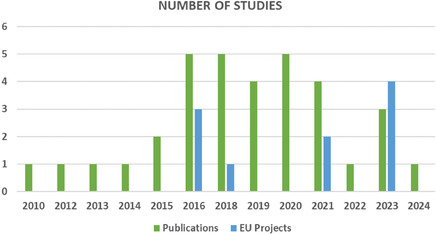
Number of LCC publications and EU‐projects per year between 2010 and 2024.

Within the European context, eight EU‐funded projects were identified, mostly focused on development of cost‐efficient materials. However, most of these projects did not provide information regarding cost analysis. One project (AUTOSUPERCAP) resulted in a publication already identified via the academic portals. Four other projects (POSEIDON, GREENCAP, MUSIC, and HEDAsupercap) started in 2023. As of 2024, no additional European projects on this topic have been identified. **Figure** **6** illustrates the volume of LCC‐related literature published since 2010.

#### 3.2.1 Goal and Scope

The goal and scope defined in LCC studies sets the framework for the analysis by defining the objectives of the study and the boundaries within which the cost analysis is conducted.

SCs are often used in combination with other ESS (mainly based on lead‐acid (Pb‐acid) or lithium batteries), to leverage the advantages of both systems (here referred to as a mixed ESS). Similarly, SCs can be integrated with renewable power generation (frequently photovoltaics) to store surplus energy (here referred as a hybrid ESS (HESS)). Among the 34 reviewed articles, some consider hybridization of SCs with renewable power generation. For example, N. Luta et al.^[^ ^47^ ^]^ analyzed in 2018 the deployment of a HESS combining photovoltaic power generation with energy storage in hydrogen fuel cells (FCs) and SCs. Regarding mixed energy storage technologies, Conte et al.^[^ ^48^ ^]^ analyzed the integration of a Pb‐acid battery‐SC storage system for an electric forklift.

**Figure 7:**
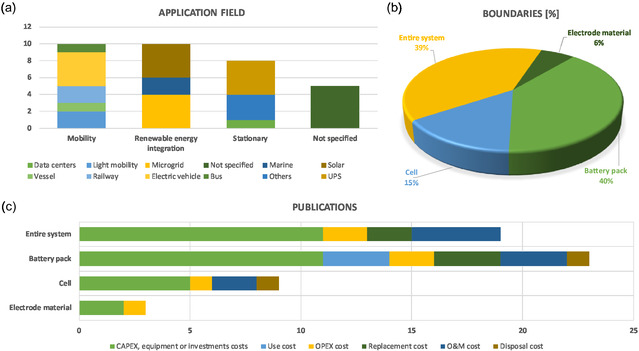
Life cycle cost literature review: a) the number of studies by application field, b) the percentage of publications by boundaries, and c) the number of publications per life cycle stages considered.

In contrast to LCA studies in this review, in the context of costing, the specific application plays a central role. **Figure** **7**a illustrates the distribution of existing literature according to application. Reflecting the growing interest in electromobility in recent years, 11 articles analyze the integration of SCs in the transport sector. For instance, Khaligh et al.^[^ ^49^ ^]^ reviewed in 2010 the state‐of‐the‐art of ESS for advanced hybrid vehicular applications, highlighting the potential use of SCs in future hybrid vehicle designs. In 2019, Wieczorek et al.^[^ ^50^ ^]^ carried out a cost comparison of different electric city bus configurations such as battery‐only or SC‐only storage as well as mixed ESS based on SC and battery ESS. The results showed a 36% reduction of the initial costs and up to a 29% reduction of operational costs when using the mixed ESS compared to battery‐only storage. Similarly, Morandin et al. studied in 2015 the effects of substituting a diesel‐based watercraft propulsion technology with a SC‐based electric propulsion system used in Venice, and found the latter to be a more attractive solution in terms of both investment and operating costs.^[^ ^51^ ^]^

Beyond electromobility, 10 studies have focused on the application of SCs to support renewable energy systems. In particular, in 2021 Kumar et al.^[^ ^52^ ^]^ carried out an economic comparison between a LIB and a SC used as ESS for a wave energy converter hourly dispatching scheme. They found that the ESS cost can be reduced by ≈61% by using the SCs instead of the LIB for this application. Similarly, in 2018 Ayodele et al.^[^ ^53^ ^]^ studied the integration of a SC in a battery/PV system for remote agricultural farm power application with the aim to increase battery lifetime and thus to reduce life cycle cost.

In other stationary applications, in 2023 Yousef Pourjamal^[^ ^54^ ^]^ published a comparative study of the techno‐economic performance of various energy storage solutions (including SCs) for fast‐acting grid balancing, analyzing different frequency control markets. Within the Nordic power system analysis, SCs were found to be the most favorable solution in terms of financial returns for fast frequency reserves, where a rapid power response is required. In addition companies such as Maxwell, Eaton, and Riello, have published cost analyses of their SC‐based systems, demonstrating cost savings for uninterruptible power supply (UPS) applications compared to LIBs.^[^ ^55^, ^56^ ^]^

Overall, cost analyses of SCs are typically performed in comparison to the dominant energy storage technology at a given time in each application. In this context, due to their excellent response to high‐power demands, SCs could potentially replace LIBs or Pb‐acid batteries in applications where short bursts of power are required, offering the advantage of extended lifespan and, consequently, reducing maintenance costs. Nonetheless, each application must be analyzed individually, as operational conditions vary significantly, complicating direct comparisons.

As with LCA studies, cross‐comparison of results from different LCC studies is not straightforward, and a broad and heterogeneous range of system boundaries was found. While some studies are limited to calculation of electrode‐material costs, others present aggregated costs at the pack level. Likewise, the FUs vary between studies, with results expressed per kW, kWh, kW‐year, year, and km. This discrepancy is largely attributed to differences in boundaries, applications, and life cycle stages considered.^[^ ^57^ ^]^

A clear definition of system boundaries in a LCC model is essential to clarify which elements are included in the cost assessment. Boundaries can be limited subcomponent production at cell level or may also encompass the production and use of full modules of SCs within a specific application. In this sense, Figure 7b illustrates the number of research studies classified according to their defined boundaries. Four boundary levels were defined. Firstly, “electrode materials”, referring to the analysis of individual cell components; “cell level”, referring to the analysis of a full SC; “battery pack”, consisting of several cells connected in series and/or parallel; and “entire system”, integrating energy storage with application‐specific components, (e.g., an electric vehicle in mobility applications or a full battery ESS in stationary applications). It should be noted that the electrode‐material level does not represent a fully functioning SC, but is included as a component‐level cost assessment to capture material‐related contributions. System boundaries are properly defined at the cell, battery pack, and complete system levels while maintaining the electrode‐material level to provide insights into material‐related costs. Of the reviewed literature, ≈9% (three studies) analyzed electrode production costs, 17% (six studies) focused on single‐cell costs, 37% (13 studies) examined battery pack manufacturing costs, and another 37% (13 studies) assessed costs at the system level, including the use phase.

Among studies focused on electrode materials, attention has been placed on the synthesis of AC for electrode processing. In this sense, Weinstein et al.^[^ ^58^ ^]^ described the costs of different carbon production methods, while Wang et al.^[^ ^40^ ^]^ analyzed the cost and environmental impacts of production of AC. Five articles were found to conduct a LCC on the cell level. Among them, Mao et al.^[^ ^59^ ^]^ relied on the economics of a 3000 F–2.7 V Maxwell SC to size the storage system and calculate installation and replacement costs. Similarly, Pourjamal et al.^[^ ^54^ ^]^ conducted a cost analysis of a 3200 F–2.85 V SC from Skeleton Technologies. Several studies addressed broader system boundaries. For instance, Gaetani‐Liseo et al.^[^ ^60^ ^]^ presented the investment costs of a SC module and, by simulating the load profile of the respective application, calculated the levelized cost of energy (LCOE) of the system. In 2010, Khaligh et al.^[^ ^49^ ^]^ conducted a review of both the technical and economic performance of SCs at the pack and system levels for hybrid electric vehicles (HEVs). The article summarized structures, characteristics, and costs of ESS topologies, as well as comparisons of typical market‐available HEVs. Beyond pack costs, the study also assessed the entire vehicle system. Gbadegesin et al.^[^ ^61^ ^]^ compared the levelized cost of HESS for different technology configurations (SCs with Pb‐acid batteries, LIBs, or hydrogen FCs), and Mongird et al.^[^ ^62^ ^]^ analyzed the technical and economic aspects of ESS at the pack level, considering different types of technologies (such as UCs) as well as power electronics balance‐of‐system components.

With regard to system boundaries, more detailed information is typically available at the battery pack or full system level. However, differences among manufacturers result in significant variations in product design, making cost comparisons at the cell or pack level difficult. At the system level, costs also include additional components required for operation, which vary depending on the application and technical specifications. This further complicates direct cost comparisons.

#### 3.2.2 Data Sources

Similarly to LCA, understanding the quality of data for LCC is crucial to ensure reliability of results. Within the 34 articles analyzed, a majority relied heavily on secondary data. Specifically, in 2018, Luta et al.^[^ ^47^ ^]^ employed the Homer Pro software to model, simulate, and optimize system configurations, as well as to estimate installation and operational costs over the system's lifetime. Other popular simulation software, such as MATLAB, has been used by Wang et al.,^[^ ^63^ ^]^ who developed a multistrategy snake optimizer for calculating the minimum cost of a hybrid system comprising wind and photovoltaic power generation combined with batteries and SCs. Similarly, Kumar et al.^[^ ^52^ ^]^ used the MATLAB Simulink environment to optimize the cost of an ESS for a wave energy converter with a SC bank, explicitly accounting for both cycling and calendar aging.

Other literature sources have collected and provided primary data, such as Mao et al.,^[^ ^59^ ^]^ who conducted an economic evaluation of energy storage solutions for an industrial microgrid employing Maxwell BCAP3000 SC cells. In addition, in 2020, Saarentausta et al.^[^ ^64^ ^]^ developed a technical and economic model of a mixed ESS based on LIBs and SCs, drawing on data from LIC manufacturers such as LS Mtron, Skeleton, Sech, Maxwell, Musashi Technologies (formerly JM energy), and AOWEI. Similarly, Mongird et al.^[^ ^62^ ^]^ conducted a comparison of different energy storage technologies (including UCs) after direct interaction with several stakeholders. Finally, manufacturers such as Maxwell, Eaton, and Riello have also published cost analyses of their own SC‐based systems.^[^ ^55^, ^56^ ^]^

#### 3.2.3 Cost Assessment Methodology

In LCC studies, the choice of assessment methodology depends on the study's goal and determines the cost categories of interest, such as capital expenditures (CAPEX), operational expenditures (OPEX), use‐phase costs, operation and maintenance (O&M) costs, and disposal costs. CAPEX refers to the initial costs of a product, considering the various expenses associated with product implementation. In this sense, Mongird et al.^[^ ^65^ ^]^ reported a cost breakdown of the CAPEX in a stationary application, distinguishing between elements such as the power control system, balance of plant (BOP), and construction and commissioning, among others. OPEX, on the other hand, encompasses recurring costs associated with repair, inspection, and other operational tasks.^[^ ^62^ ^]^ However, the reviewed articles employ different terminology for cost categories (e.g., CAPEX, OPEX, O&M, and replacement). For the sake of rigor, costs are reported as originally presented in each study, rather than being homogenized, as doing so could risk mixing nonequivalent categories and misrepresenting the original data. Figure 7c displays the distribution of studies classified according to this methodology as well as to the system boundaries.

#### 3.2.4 Cost Assessment of SCs

To discuss the cost assessment of SCs, it is necessary to compare findings across the literature. Such comparison should provide a basis for drawing conclusions on SC costing. However, this is not a straightforward task, as studies differ in terms of system boundaries and cost categories.

In this review, costs have been classified according to the main components of the system: electrode material, cell, battery pack, and entire system. This classification allows for a more specific analysis of costs associated with each part of the system and facilitates understanding of how costs are distributed across different levels of the device. A detailed analysis of CAPEX, OPEX, O&M, and use‐phase costs has been assigned to these components, enabling a comprehensive view of the economic dynamics of SCs.

It is noteworthy that, although the classification focuses on system components, these costs are intrinsically linked to life cycle stages such as material extraction, production, use, and recycling. For instance, CAPEX costs associated with the cell and battery pack are typically incurred during the production stage, as they involve the manufacturing and assembly of these components. Similarly, OPEX costs for the complete system are more relevant during the use stage, when the SCs are operational, functioning, and providing energy. Regarding O&M costs, these are primarily distributed in the use phase, as they are associated with the ongoing operation and management of the SCs, such as monitoring and maintaining the cells, battery pack, and the entire system. These costs reflect the operational needs of the device during its active lifespan. Thus, classifying costs by component is not incompatible with a life cycle perspective. Rather, it provides a more granular view of cost structures while still considering their relationship to different stages of the life cycle. This methodology is particularly useful for evaluating economic impacts across a product's phases without losing sight of interactions between components and stages.

At the electrode‐material level, Wang et al.^[^ ^40^ ^]^ conducted a study in 2022 analyzing both the investment costs of AC production and the operational costs of the production plant, assessing the economic feasibility of AC synthesis. In the analysis, the authors found a breakeven point of 16.97 USD kg^−1^.

In 2024, Luanwithi et al.^[^ ^44^ ^]^ investigated the economic impacts of AC made from oil palm leaves through hydrothermal carbonization and chemical activation. The cost assessment was made based on experimental data, generating input data for the mass and energy balance, including processing temperature and AC yield. They compared the investment costs for different scales of production, ranging from 720 to 1080 tons per year of oil palm leaves. All scenarios resulted in positive net present values (NPV), with minimum selling prices ranging from 10.50 to 13.40 USD kg^−1^. These studies highlight how production methodology influences AC cost.

At the cell level, EIT InnoEnergy^[^ ^66^ ^]^ reported in 2020 CAPEX cost of 5000–10,000 $ kWh^−1^ for SCs, compared with 100–1000 $ kWh^−1^ for batteries. Pourjamal et al.^[^ ^54^ ^]^ (2023) analyzed both CAPEX and OPEX of SkelCap cells, assuming a 10‐year lifetime, with values of 19.5 € Wh^−1^ (CAPEX) and 0.136 € kW^−1^ yr^−1^ (OPEX). Mao et al.^[^ ^67^ ^]^ reported CAPEX and O&M costs for Maxwell BCAP3000 SCs, with unit capital costs of 10,000 USD kWh^−1^ and replacement costs of 3000 USD kWh^−1^. Lastly, Wang et al.^[^ ^63^ ^]^ considered CAPEX, O&M, and disposal costs (including salvage and EOL) in a hybrid system comprising wind turbines, PV arrays, batteries, and SCs. Their cost‐optimization model identified a total cost reduction potential of ≈4.5%.

At the battery pack level, 11 studies analyzed CAPEX costs. In particular, Carter et al.^[^ ^68^ ^]^ evaluated the investment costs of two 30‐cell SC packs (1700–2600 F) for use in an electric vehicle. While SCs effectively reduced peak battery currents, at least a 50% extension of battery lifetime was necessary for cost‐effectiveness. Wieczorek et al.^[^ ^50^ ^]^ studied a mixed battery pack comprising 222 SC cells in series and 13 or 38 cells in parallel, integrated with LIBs in an electric city bus. They found that including SCs reduced both battery size and initial ESS price, as well as operational costs. Other studies make use of simulation tools to assess the associated costs of the use‐phase, evaluating the influence of parameters such as the depth of discharge (DoD) and the lifetime of the system.

Kumar et al.^[^ ^52^ ^]^ analyzed the economically optimal DoD for an SC system, finding that full utilization (100% DoD) resulted in the lowest cost.

A distinction must be made between OPEX, replacement, and O&M costs, as these terms are often used in overlapping contexts but have different meanings. For example, Kim et al.^[^ ^69^ ^]^ included fixed O&M costs under OPEX together with fuel and electricity expenses, and reported that electricity and fuel consumption were the dominant contributors. In contrast, Gbadagesin et al.^[^ ^61^ ^]^ treated O&M and replacement costs separately, showing that replacement costs over the project lifetime outweighed O&M. Finally, few articles consider the disposal costs. For instance, Riello assumes the disposal cost of a 5 kW SC as 85 €.^[^ ^55^ ^]^

At the entire system level, the literature primarily focuses on CAPEX and investment costs.^[^ ^61^, ^62^, ^69^ ^]^ As an example, in 2016, Tomczyk et al.^[^ ^70^ ^]^ implemented a model to assess the cost‐effectiveness of railway electrification, which included both vehicle purchase and maintenance costs. Notably, among the 13 studies analyzing entire‐system costs, none considered disposal.

It can be seen that LCC results vary substantially depending on system boundary and application, which also affects the choice of FU. At the electrode‐material level, costs are usually expressed per kilogram of production, reflecting raw material inputs. At the cell and battery pack levels, OPEX and use‐phase costs become relevant, often expressed per year or per kW over operating time. For complete systems, O&M and replacement costs dominate, alongside other subsystem costs tied to the application (e.g., BOP). Thus, careful alignment of system boundaries with the intended application is essential to ensure that the most relevant cost factors are captured.

Given the wide range of applications, system boundaries, and cost categories, direct comparison of results across studies is not feasible. Nevertheless, most assessments compare SC costs against benchmark energy storage technologies to evaluate economic viability, which ultimately determines feasibility for market adoption. In this sense, and as it happens with LIBs, system degradation of SCs and hybrid Caps is strongly influenced by operating conditions (e.g., temperature, DoD), which in turn affect performance, lifetime, and O&M costs. Some authors have analyzed these effects, such as Song et al.^[^ ^57^ ^]^ who modeled monthly degradation costs of a HESS under varying operating temperatures, finding that cold winter months (below 0 °C) were associated with higher degradation costs. In a similar way, Gbadegesin et al.^[^ ^61^ ^]^ investigated the effect of system degradation on energy output and replacement costs over a 20‐year period. The authors found that neglecting the effects of system degradation leads to potentially high overestimations of lifetime energy output, highlighting the importance of taking into consideration performance decay and system degradation in project costing.

Another important factor is the year of calculation, as system and material costs fluctuate with demand, geopolitical events, and social factors. Consequently, results from studies conducted in different periods are not directly comparable. It may be possible, nevertheless, to make cost predictions, as in the case of Mongird et al.,^[^ ^62^ ^]^ who in 2019 projected the CAPEX and O&M costs of entire systems for 2025. In a similar way, EIT InnoEnergy^[^ ^66^ ^]^ published historical and projected cost trajectories for SCs between 2005–2030. However, such projections must be treated with caution, as external factors, such as economic crises or global supply chain disruptions, can dramatically alter material and component costs, introducing significant uncertainty into long‐term forecasts.

Overall, the available LCC studies on SCs remains fragmented and strongly dependent on methodological choices. The lack of harmonized system boundaries and FUs prevents meaningful comparison across studies and with alternative storage technologies. Moreover, the cost‐effectiveness of SCs is highly application‐dependent: while they may appear less competitive when assessed in terms of cost per kWh, they can outperform conventional batteries in cost per kW or per cycle in high‐power, short‐duration applications. This reflects the importance of the application in the cost analysis. Despite this, few works systematically integrate such application‐driven metrics or consider degradation, recycling, and second‐life scenarios, which clearly influence lifetime costs. Addressing these gaps through standardized and application‐oriented LCC methodologies would enable more robust cost evaluations and support decision‐making for the large‐scale deployment of the technology.

## 4 Discussion and Conclusions

In this article, a methodological review of available environmental (LCA) and cost (LCC) assessment studies for SCs has been carried out. The search was made through specific search engines and keyword strings for each type of study, identifying a total of 24 LCA and 34 LCC scientific research articles. At the European project level, a total of 15 and 7 sources for LCA and LCC, respectively, were found. Despite their long‐standing development, SC technologies have only recently become subject to more systematic sustainability assessments. Environmental assessments have increased since 2019, whereas cost assessments have been significant since 2016. Nonetheless, the total number of studies remains limited, and several data gaps persist.

Within the available literature, this review analyzed key aspects of LCAs, including goal and scope (system boundaries), data sources, impact assessment methodologies, and total environmental impacts. As is common in energy technologies, climate change potential was the most frequently assessed impact category, whereas relatively few studies examined issues of resource criticality in SCs. Such analyses could provide important insights into trade‐offs both between different Cap technologies (e.g., LIC vs. SIC) and in comparison, to benchmark technologies such as batteries. Furthermore, many assessments have concentrated on electrode‐material production from a cradle‐to‐gate perspective, leaving significant uncertainty regarding the use phase, where technical performance factors (e.g., energy density or charge/discharge efficiency) strongly influence environmental outcomes. In the case of LCCs, the system applications, boundaries, and life cycle stages have been at the center of the analysis. While investment costs are frequently assessed, disposal costs for SC technologies are rarely addressed. Moreover, for cost analyses, the year of publication is a critical factor, as technology maturity, user demand, and broader socioeconomic conditions can substantially influence results. These dynamics complicate direct comparisons across studies and highlight the need for harmonized approaches to both environmental and cost assessments of SCs.

In general, the screening of environmental and economic assessments suggests that, due to the heterogeneous system boundaries used in the literature, it is currently unfeasible to estimate average environmental and cost performance levels for SCs. The broad variety of applications, technology types and system levels, combined with the limited number of studies providing sufficient detail in the analysis (often related to very novel systems), prevents drawing meaningful and sensible conclusions about the impacts of SCs at this stage. The available results may inform discussions of specific case studies, but they offer little insight into the broader picture of SC technologies. Thus, it remains unclear how generic SCs technologies may perform from an environmental and economic point of view. Further research is therefore necessary to establish a more comprehensive understanding of their sustainability implications. Moreover, ongoing research aims to develop next‐gen SCs such as LICs and SICs.^[^ ^71^ ^]^ Because they are still at their infancy, the uncertainty surrounding these emerging systems leads to a profound lack of understanding about their potential environmental implications, reflected in the small body of literature available for this type of technologies. This highlights a knowledge gap about the potential sustainability trade‐offs that may arise from their implementation and about their advantages and disadvantages compared to benchmark systems, which needs to be addressed to ensure sustainable development.

It is noteworthy that most studies provide fully or partially disclosed LCIs, which enhances transparency and facilitates reproducibility and traceability of results. This fact would also enable potential reconstruction, harmonization and comparison between different systems under a common framework and set of key parameters, thereby contributing to a clearer picture of environmental and economic impacts of SCs.

The challenges encountered in this review, particularly those hampering cross‐study comparison and the extraction of a general picture of environmental impacts and cost, also highlight the need for a follow‐up study focused on the unification of boundaries and subsequent recalculation of cost and impacts. This can be achieved by adapting published life‐cycle inventories to a common set of parameters and boundaries (e.g., electricity mixes and system components) and by using a single, well‐defined FU as a shared basis for comparison. Distinguishing production scales and technology‐readiness levels before interpreting results is essential, since both directly influence outcomes. Because few studies analyze comparable system levels (e.g., active materials, electrodes, full devices or specific applications), limitations and uncertainty will remain even after harmonization; expanding the evidence base with new assessments and reproducible models therefore remains relevant. In parallel, studies should align goal and scope with system boundaries, for instance, conducting cradle‐to‐gate assessments for materials or process screening, and cradle‐to‐grave evaluations for complete devices or applications that include use‐phases and EoL. Service‐based FUs are preferable for device‐and application‐level studies, with associated operating conditions and power‐delivery constraints reported explicitly. Transparency on electricity mix (country and year), background database or software, and production scale (lab, pilot, and industrial), together with the use of prospective grid scenarios where relevant, will improve comparability. Scenario and sensitivity analyses should cover electricity mix, scale‐up yields and material or heat recovery, and EoL modeling to strengthen representativeness. For cost assessments, defining a clear scope (CAPEX, OPEX, replacements, disposal/EoL) is essential, including timestamps and price assumptions (year, geography, and currency), providing normalized values (€ kW^−1^, € kWh^−1^) alongside total cost of ownership (TCO) over a defined lifetime. Testing duty‐cycle and degradation‐coupled scenarios should also be incorporated. Collectively, these measures could enable more robust, comparable assessments to better guide the sustainable design and deployment of SCs.

## Conflict of Interest

The authors declare no conflict of interest.

## Author Contributions

**Fatemeh Bahmei**: conceptualization, methodology, validation, formal analysis, investigation, writing—original draft, visualization. **Amaia Saenz de Buruaga**: conceptualization, methodology, validation, formal analysis, investigation, writing—original draft, visualization. **Sebastián Pinto Bautista**: conceptualization, methodology, validation, investigation, writing—original draft, visualization. **Javier Olarte**: validation, writing—review & editing, supervision. **Jon Ajuria**: validation, writing—review & editing, project administration. **Alberto Varzi**: validation, writing—review & editing. **Marcel Weil**: conceptualization, methodology, validation, writing—review & editing, supervision. **Fatemeh Bahmei**, **Amaia Sáenz de Buruaga** and **Sebastian Bautista** contributed equally to this work.

## Declaration of Generative AI and AI‐Assisted Technologies in the Writing Process

Statement: During the preparation of this work the author(s) used ChatGPT in order to improve readability and language. After using this tool, the author(s) reviewed and edited the content as needed and take(s) full responsibility for the content of the publication.
